# In vitro inhibition of HUVECs by low dose methotrexate – insights into oral adverse events

**DOI:** 10.1186/1746-160X-10-19

**Published:** 2014-05-22

**Authors:** Tobias Annussek, Thomas Szuwart, Johannes Kleinheinz, Cathrin Koiky, Kai Wermker

**Affiliations:** 1 Department of Cranio-Maxillofacial Surgery, University Hospital of Muenster, Research Group Vascular Biology of Oral Structures (VABOS), Waldeyerstr.30, Muenster 48149, Germany; 2 Department of Cranio-Maxillofacial Surgery, Fachklinik Hornheide at the Westphalian Wilhelms University of Muenster, Muenster, Germany

**Keywords:** Oral health, Antirheumatic drugs, Methotrexate, Endothelial cells, In vitro, Wound healing

## Abstract

**Background:**

With socio-economic changes, dentists and maxillofacial surgeons are more and more faced with medically compromised patients. Especially, the admission of antirheumatic drugs has increased remarkably. So dentists and maxillofacial surgeons should be aware of related adverse reactions that affect the craniofacial region. To identify possible cellular effects of disease modifying antirheumatic drugs (DMARDs) we investigated the influence of methotrexate (MTX) on human umbilical vein endothelial cells (HUVECs).

**Methods:**

HUVECs were incubated with various concentrations of MTX, corresponding to serum concentrations found in rheumatoid arthritis (RA) patients. The effect of MTX on cell proliferation, differentiation as well as mitochondrial activity was measured by use of immunostaining, cell counting and 3-(4, 5-dimethylthiazol-2-yl)- 2, 5-diphenyltetrazolium bromide (MTT) assay.

**Results:**

All samples incubated with MTX (1-1000 nM) showed significantly decreased cell viability when compared to controls. Cells were less proliferating, but did not lose their ability to synthesize endothelial proteins. A slight dose dependency of inhibiting effects was demonstrated. The observed differences between control and sample groups were rising with longer duration.

**Conclusion:**

Because of the crucial role of endothelial cells and their precursor cells in wound healing, a negative influence of MTX on oral health has to be supposed, correlating to clinical observations of adverse reactions in the oral cavity, such as ulcerative or erosive lesions.

## Background

Within the rising of expectancy of life dental and maxillofacial practitioners are more and more faced with medically compromised patients [1]. These patients represent a possible risk in oral healthcare, especially concerning wound healing after surgical procedures like implant insertion, augmentation, open reduction and internal fixation (ORIF) of fractures or accidental lesions [2,3]. Within the most frequent diseases, inflammatory diseases and thus the rheumatoid arthritis (RA) is statistically dominant with a prevalence between 0.5-1% in developed countries and a female/male ratio of 3:1 [4,5]. An early use of pharmacological substances, the so called disease modifying antirheumatic drugs (DMARDs), is the key pattern in RA therapy as recommended [6]. Since more than two decades, low dose methotrexate (MTX, 5-25 mg/weekly) has been established as first-line therapeutic agent [7] and has thus been widely used drug in RA therapy [8]. Currently, the American College of Rheumatology (ACR) and the European League Against Rheumatism (EULAR) have recommended even higher dosages [9,10]. Previous to low dose therapy in RA, MTX was used in oncology at higher dose as antineoplastic agent [11]. Most of our knowledge about mechanism of action, pharmacokinetics and side effects of MTX is derived from high dose therapy, whereas the precise mechanism of antirheumatic action has not yet been understood in detail [12]. MTX and its metabolites (MTX glutaminated) inhibit the dihydrofolate reductase (DHFR), thymidylat synthase and 5-aminoimidazole-4-carboxamide ribonucleotide (AICAR) transformylase, which leads to accumulation of extracellular adenosine. This extracellular adenosine accumulation has been found out to mediate the anti-inflammatory effect of MTX [13,14]. Side effects are thought to be related to purine and pyrimidine synthesis inhibition as well as folic acid antagonism [15]. Oral events related to MTX are reported in the literature, where the risk of developing oral lesions was higher in RA patients receiving MTX therapy than in those receiving the drug and most frequent events were ulcerative und erosive lesions localized at the alveolar mucosa and tongue [16-19].

Even if the perioperative use of MTX in general or orthopedic surgery seems to be save [20], the effect on oral and maxillofacial surgery remains uncertain. Although, it was already shown that oral mucosal healing is different to healing of on skin areas [21], angiogenesis and thus endothelial cell differentiation and proliferation are a key pattern in general wound healing by formation of new blood vessels, providing migration of leucocytes, transportation of oxygen and secretion of biologically active substances [22-24]. Human umbilical vein endothelial cells (HUVEC) have been widely used and generally accepted for analysis of wound healing in vitro [25,26]. For this reason we studied the in vitro effect of MTX on proliferation, mitochondrial metabolism and differentiation of human umbilical vein endothelial cells.

## Methods

### Cell culture

According to the method of Jaffe et al., HUVECs were obtained from the veins of human umbilical cords of healthy donors [27]. The whole processing was performed under sterile conditions after disinfection of the cord by use of 70% ethanol (AppliChem GmbH, Darmstadt, Germany). Firstly areas of the cord, which were manipulated by clamps, were cut off. The umbilical vein was identified by inspection, cannulated with a blunt 14 gauge needle and rinsed with phosphate buffered saline (PBS-Puffer Dulbecco, Biochrom AG, Berlin, Germany). After eliminating PBS and obturating the other side of the cord, the vein was filled with collagenase 0.05% (Roche Diagnostics GmbH, Mannheim, Germany) and incubated for 10 minutes at 37°C. To facilitate cell detachment, the cord was massaged and squeezed after incubation time. The vein was perfused with endothelial cell growth medium (Promocell, Heidelberg, Germany) and the cell containing solution was collected. Cells were harvested by 7 minutes of centrifugation at 1200 rpm. The platelet was resuspended with endothelial cell growth medium and seeded into previously 0.5% gelatine-coated culture dishes at 37°C in a humidified atmosphere with 5% CO_2_. Until reaching confluence, the medium was changed every second day. After HUVECs were second time passaged by incubating the confluent monolayer with 0.05% trypsin/0.02% EDTA solution and replating, cells were taken for experimental procedure. They were seeded into 24 well-plates (TPP AG,Trasadingen, Switzerland) at concentrations of 1×10 cells per well and incubated for two more weeks with a solution of culture medium supplemented with MTX at concentrations ranging from 0 nM (control) to 1000 nM, according to MTX concentrations found in patients serum [28]. Additionally cells were cultured under the same conditions on culture dishes of 87.2 mm diameter (NUNC, Langenselbold, Germany). Control samples, cultured in medium without MTX, were created for each point of investigation. An exponential dilution series of cells with 1 × 10 cells per well as a starting point, was used to check the method and to ensure that the cells used for experiments were in the exponential growth phase. Since the beginning of the experimental procedure cell morphology was monitored daily by phase-contrast light microscopy. All samples were done independently in double triplicates (total n = 516). Medium was changed twice weekly. The experimental design was approved by the Ethics Committee of the Faculty of Medicine, University of Muenster. Written informed consent for participation in the study was obtained from all donors of umbilical cords.

### Cell counting

To determine the rate of cell proliferation in MTX and control samples a digital photo (NIS Elements 2.20, Nikon Instruments Inc., Melville, NY, USA) of each well was taken on days 1, 3, 6, 8, 10 and 14 after the beginning of the experimental procedure. Method was standardized, using a Java-based image processing program (Image J Cell Counter, National Institute of Health, USA). Two different, blinded examiners were instructed to count the visualized, living cells per unit area. The proliferation rate was calculated by the ratio of living cells at starting point (day 0), compared to trial days 1 to 14. Interrater reliability was tested, calculating Cohen’s kappa by using SPSS software (version 15.0; SPSS Inc., Chicago, Illinois, USA).

### Viability assay

According to the above described protocol the viability, more precise, the mitochondrial activity of living HUVECs, was measured by performing a 3-(4,5-dimethylthiazol-2-yl)2,5- diphenyl-tetrazolium bromide (MTT) assay (SigmaAldrich Co., St. Louis, MO, USA). The assay, as described by Pannecouque et al., was slightly modified and done for all samples (1- 1000 nM) including control without MTX addition [29]. For this, cells were incubated for one hour with 1 ml MTT solution (0.25 mg/ml medium) at 37°C in a humidified atmosphere with 5% CO_2_. Within incubation time living cells metabolised the MTT to formazan crystals. Thereafter, medium was aspirated and 200 μl of propanol was added to lyse the cells and dissolve the released formazan crystals (n = 180). To complete dissolution of the formazan salts, plates were placed on a vibrating platform shaker for 10 min. The extinction value was measured at a wavelength of 570 nm using an enzyme-linked immunosorbent assay reader (μQuant, Biotek instruments, Bad Friedrichshall, Germany).

### Immunostaining

To characterize differentiation of HUVECs while MTX addition, the expression of extra cellular matrix proteins (e.g. CD31, von Willebrand factor, alpha smooth muscle actin, SigmaAldrich Co., St. Louis, MO, USA) was measured. Additional culture dishes (0-1000 nM MTX) were used for immunostaining. When control samples reached confluence, the expression of CD 31 and von Willebrand factor (vWF) as well as alpha smooth muscle actin (α-SMA, negative control) was determined. Cells were washed twice with phosphate buffered saline and fixated for 20 minutes at −20°C. One hundred microL of blocking solution (CANDOR Bioscience, Wangen, Germany) was used for each sample and a period of 15 minutes. According to the manufacture’s instruction, primary antibodies (CD 31, vWF, α-SMA) as well as secondary antibodies (Alexa Fluor 488, Life Technologies, Carlsbad, Canada) were incubated for 60 minutes at 37°C (n = 30).

### Statistical analysis

All statistical analyses were performed by a statistician using SPSS software (version 16.0; SPSS Inc., Chicago, Illinois, USA). To distinguish between groups of different MTX- concentrations (0-1000 nM) we performed analysis of variance (ANOVA, post hoc Tamhane T2 – test). Interrater reliability was tested calculating Cohen’s kappa.

## Results and discussion

### Cell differentiation

**Figure 1:**
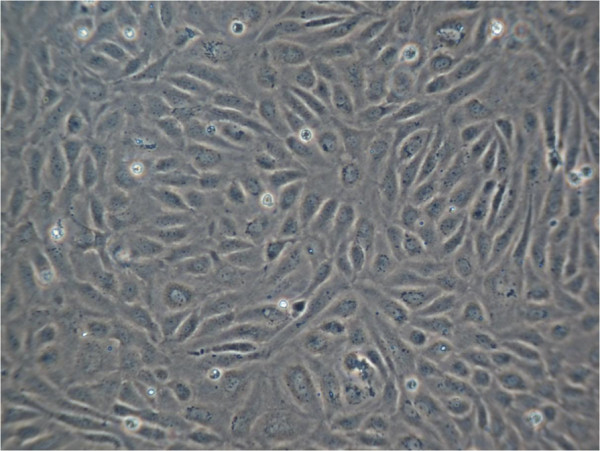
HUVECs phase light microscopy 10×, day 10 typical endothelial morphology, confluent monolayer without MTX addition (control).

**Figure 2:**
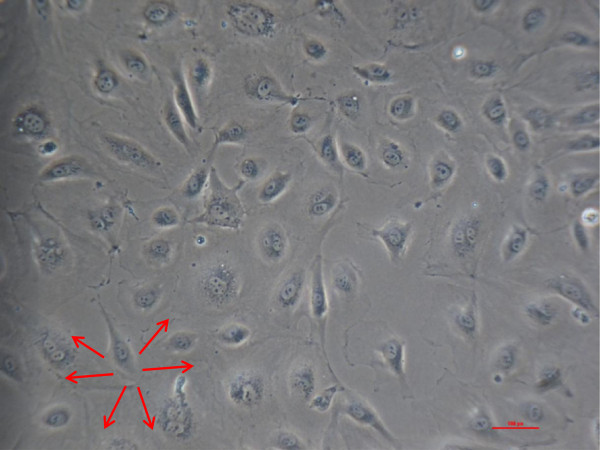
**HUVECs phase light microscopy 10x, day 10, missing of typical endothelial morphology, no confluent monolayer at 1000 nM MTX.** Red arrows mark exemplary diminished cell-cell interaction.

**Figure 3:**
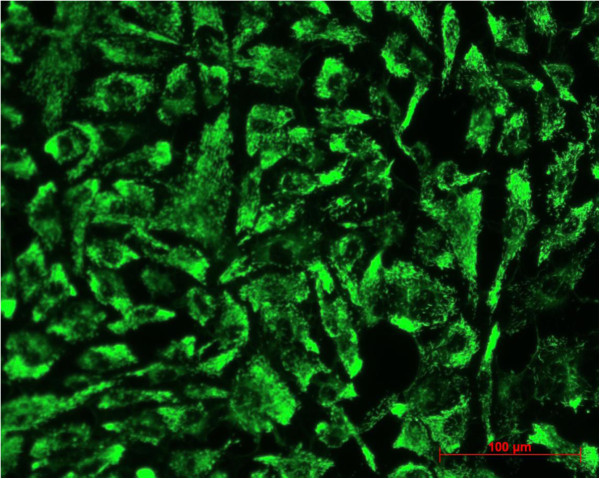
CD 31 expression at control after 10 days.

**Figure 4:**
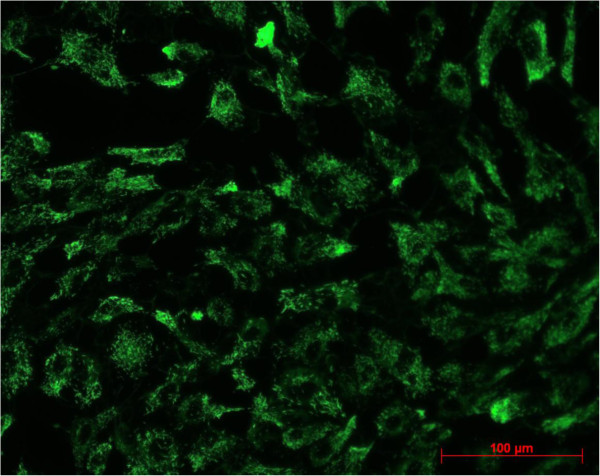
CD 31 expression at 1000 nM MTX after 10 days of incubation.

**Figure 5:**
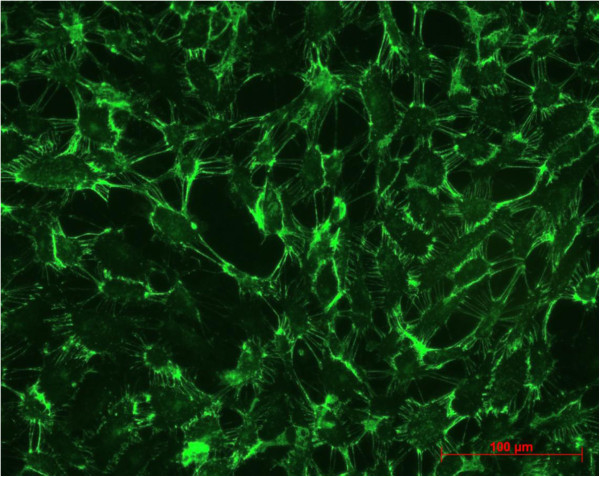
vWF expression at control group after 10 days.

**Figure 6:**
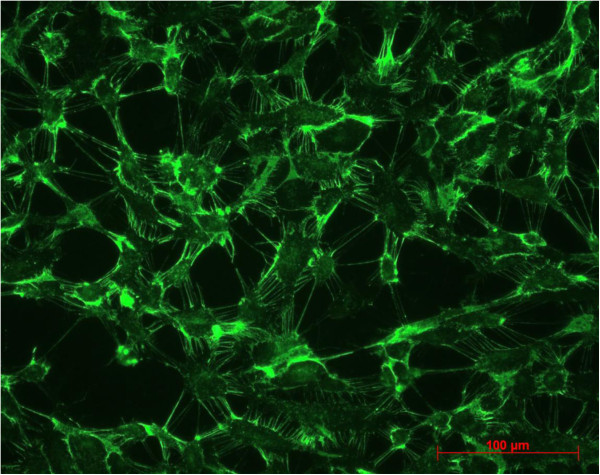
**vWF expression at 1000 nM MTX after 10 days of incubation.** Diminished cell-cell interaction could be seen by high frequency of dark gaps between single cells.

Between second passage and the end of experimental procedure the typical endothelial morphology of HUVECs was seen by daily phase light microscopy (Figure 1). At the starting point they were tightly packed, uniformed, polygonal cells, reaching a stable, confluent monolayer. Mean diameter of HUVECs was 18.72 ± 0.91 μm, assessed by electronic particle counter (CASYI®, Schärfe System GmbH, Reutlingen, Germany). Within the first three days of investigation no differences between MTX and control groups was observed. Thereafter, cell behaviour and morphological characteristics began to differ, without affecting cell differentiation in general. With increasing MTX concentration and longer duration of the trial, cells were proportionally less tightly packed and not that uniformed, polygonal when compared to control samples (Figure 2). Mean diameter increased slightly to 19.57 ± 0.79 μm without statistically significant difference compared to starting point (p = 0.692, t-test). However, expression of epithelial proteins was not affected by MTX addition in general. Cells were still synthesizing CD31 and vWF as proven by immunostaining (Figures 3 and 4). Nevertheless, diminished cell-cell interaction was seen at all MTX concentrations but without remarkable proportionality to rising MTX concentration when compared to control (Figures 5 and 6). All samples of α -SMA expression were negative (data not shown).

### Cell proliferation

Table 1 presents the measured cell number per unit area for every MTX concentration on days 1, 3, 6, 8, 10 and 14. Concerning proliferation of endothelial cells after administration of different concentrations of MTX we observed no differences in relative cell number neither between different MTX levels nor compared to control group without MTX addition. From day 6 on, a significant inhibiting effect between groups of various MTX-concentrations was noticed, however not reaching the defined level of significance (p > 0.05, ANOVA). On day 6, difference concerning the relative cell number was highly significant with p < 0.001 between controls (no MTX) and MTX-concentrations of 10, 100 and 1000 nM. Between control and 1 nM MTX the difference was not yet significant (p = 0.056). Between 1 nM MTX and other MTX- concentrations (10, 100 and 1000 nM), inhibitory effect also reached statistical significance (p-values 0.026, 0.003 and 0.006, respectively). From day 8 on, the inhibition of cell proliferation as measured by cell counting was highly significant (p < 0.001) for all four administered MTX-concentrations compared to control. For all measurements, interexaminer reliability was high with Cohen’s kappa κ = 0.924.

**Table 1:** Results of cell count

**Cell number per unit**		**Control**	**1 nM**	**MTX-concentration**		
				**10 nM**	**10 nM**	**10 nM**
Day 1	mean	13.50	17.83	28.67	25.50	33.17
	SD	4.76	5.74	13.55	9.01	7.49
	95% CI	10.48-16.52	11.81-23.86	14.45-42.88	16.05-34.95	25.30-41.03
Day 3	mean	37.58	54.02	74.01	65.83	85.17
	SD	15.72	19.22	18.24	9.04	12.07
	95% CI	27.59-47.57	33.84-74.16	54.86-93.14	56.34-75.32	72.50-97.84
Day 6	mean	159.92	110.67	69.33	63.67	70.50
	SD	47.86	15.85	19.63	7.23	10.21
	95% CI	129.51-190.33	94.04-127.30	48.73-89.94	56.08-71.25	59.78-81.22
Day 8	mean	222.04	97.51	59.83	56.17	63.67
	SD	40.72	18.08	23.09	12.70	12.31
	95% CI	192.13-247.87	78.53-116.47	35.60-84.07	42.84-69.50	50.75-76.58
Day 10	mean	221.17	68.00	31.17	33.53	36.33
	SD	51.30	30.68	7.71	16.79	6.22
	95% CI	188.57-253.76	35.80-100.20	23.08-39.25	15.88-51.12	29.81-42.86
Day 14	mean	210.92	90.83	41.17	37.83	40.83
	SD	55.25	49.06	8.09	16.52	11.48
	95% CI	175.82-246.02	39.34-142.34	32.68-49.65	20.49-55.17	28.79-52.88

SD = standard deviation; 95% CI = 95% confidence interval.

### Cell viability

**Figure 7:**
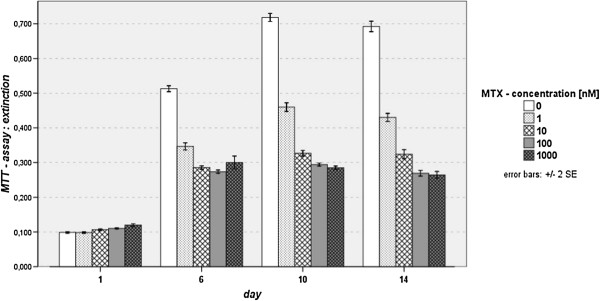
Results of MTT assay, significant dose-dependent inhibiting effects of MTX on mitochondrial activity and viability.

Within the first 72 hours of MTX incubation the mitochondrial activity of all samples (1- 1000 nM MTX) and control (0 nM MTX) measured by extinction values (MTT Assay) was relatively equal and slightly rising. With longer duration, values of the control group were increasing more rapidly with the highest extinction value on day 10 (E = 0.718). Thus, the highest mitochondrial activity was observed in the control group. While incubation with 1 nM MTX, an increased extinction value was also found however, 1.5 fold lower in the control group. The mitochondrial activity measured at 100 and 1000 nM MTX was relatively unaltered. Statistical analysis of group comparisons showed high significant differences (p < 0.001) for all four MTX groups compared to control, from day 6 on. Additionally, inhibition of mitochondrial activity was significantly less strong in the 1 nM MTX-group compared to MTX- concentrations of 10, 100 and 1000 nM (p < 0.001). These significantly dose-dependent extinction values found by use of MTT-assay are shown in Figure 7.

## Discussion

Only little interest has been focused on MTX induced oral toxicity, despite various case reports dealing with chronic oral ulceration, sore, or recurrent stomatitis during low-dose MTX therapy [30,31]. As described by Katalanzis et al., the variety of oral lesions ranges from nonhealing ulcers to destructive lymphomalike lesions [32]. Other authors presented cases of progressing necrotizing ulcerative gingivitis involving lips and oral mucosa, chronic ulcer of the hard palate and MTX therapy-related impairment of the oral mucosa in general [33]. It is thought that clinically observed side effects are signs of systemic conditions leading to direct MTX toxicity, which were tried to be reduced by supplementary folic acid treatment. However, the reduction of oral adverse events was not proofed by statistical significance [34]. Thus oral adverse events may be underestimated by dentists and maxillofacial surgeons. Deschaumes and colleges showed that oral mucosal damage is initiated by endothelial cell death [35]. For this reason, we chose endothelial cells for investigating basic healing patterns by knowing that they are not specific for the oral or facial region, but indispensable for wound healing in general. In most cases, lesions disappeared slowly compared to clearance half-life when interrupting MTX therapy [36]. Additionally to slow cellular clearance of MTX glutamates, we propose an endothelial cell mediated mechanism. Our results suggest the inhibition of proliferation, viability and mitochondrial activity of cultured HUVECs by low dose MTX. Moreover, we experimentally established a dose dependency of inhibitory effects which was missing before [37]. Other studies investigating the effect of MTX as chemotherapeutic agent suggested an inhibitory effect of endothelial cell proliferation [38-40]. As far as we know, only two other research groups investigated the effect of low dose MTX on endothelial cells with varying results. Yamasaki et al. found no inhibitory effect at concentration below 10 ^−7^ mol L^−1^, whereas Hirata and colleagues showed even inhibiting MTX action at 10^−9^ mol L^−1^[41,42]. The latter is consistent with our findings of an inhibitory effect of MTX on ECs in lower concentration. With respect to limited experimental procedure, our in vitro results are not able to represent the complexity and clinical features of LDMTX associated oral adverse events but give a hint to possible underlying mechanism with impact on maxillofacial surgery in patients undergoing LDMTX treatment. Other etiological factors as well as other cellular targets have to be evaluated carefully to clarify the nature of clinically observed lesions. Whether the impairment of oral mucosa leads to clinically significant changes in wound healing after dental surgery or not was not yet assessed because of lacking clinical data.

## Conclusion

The impairment of oral health in RA patients may be an underestimated problem [43]. Clinical observations like non-healing of oral ulcer, stomatitis or post-interventional infections can be explained by toxic effects, which are not securely avoided by folate supplementation. Neovascularisation and thus, endothelial cell function play an important role in general wound healing also in the oral cavity [44]. The present in vitro study demonstrated that even low dose MTX diminished HUVEC proliferation und mitochondrial activity in vitro suggesting a negative impact on oral and facial soft tissue. However, further investigation will be required to understand the detailed process of oral adverse events caused by low-dose MTX-therapy. Clinical studies should focus on the perioperative management and postoperative outcome in dental and maxillofacial surgery.

## Abbreviations

ACR: American college of rheumatology; AICAR: 5-aminoimidazole-4-carboxamide ribonucleotide; α-SMA: Alpha smooth muscle actin; CD 31: Cluster of differentiation 31; DHFR: Dihydrofolate reductase; DMARDs: Disease modifying antirheumatic drugs; EULAR: European league against rheumatism; HUVECs: Human umbilical vein endothelial cells; MTT: 3-(4 5-dimethylthiazol-2-yl) 2, 5-diphenyltetrazolium bromide; MTX: Methotrexate; ORIF: Open reduction and internal fixation; RA: Rheumatoid arthritis; vWF: Von Willebrand factor.

## Competing interests

The authors declare that they have no competing interests.

## Authors’ contributions

All authors read and approved the final manuscript. TA and KW established and designed the experiments. CK helped to collect the data. KW and JK analyzed the data. TA wrote the paper. JK and TS helped to drop the manuscript.
